# Serum homocysteine levels are decreased in levothyroxine-treated women with autoimmune thyroiditis

**DOI:** 10.1186/1472-6823-14-18

**Published:** 2014-03-01

**Authors:** Maciej Owecki, Jolanta Dorszewska, Nadia Sawicka-Gutaj, Anna Oczkowska, Michał K Owecki, Michał Michalak, Jakub Fischbach, Wojciech Kozubski, Marek Ruchała

**Affiliations:** 1 Department of Endocrinology, Metabolism and Internal Medicine, Poznan University of Medical Sciences, Przybyszewskiego St. 49, 60-355 Poznań, Poland; 2 Department of Neurology, Poznan University of Medical Sciences, Przybyszewskiego St. 49, 60-355 Poznań, Poland; 3 Department of Informatics and Statistics, Poznan University of Medical Sciences, Dąbrowskiego St. 79, 60-529 Poznań, Poland

**Keywords:** Homocysteine, Thyroid, Autoimmunity, Hashimoto disease

## Abstract

**Background:**

Hyperhomocysteinemia is a well-known cardiovascular risk factor and its elevation is established in overt hypothyroidism. Since some authors suggest that chronic autoimmune thyroiditis per se may be considered as a novel risk factor of atherosclerosis independent of thyroid function, the analysis of classical cardiovascular risk factors might be helpful in evaluation the causative relationship. Data concerning the impact of thyroid autoimmunity in euthyroid state on homocysteine (Hcy) level is lacking. The aim of this study was to evaluate Hcy level in context of anti-thyroperoxidase antibodies (TPOAbs) in euthyroidism.

**Methods:**

It is a case–control study. 31 euthyroid women treated with levothyroxine (L-T4) due to Hashimoto thyroiditis (HT) and 26 females in euthyroidism without L-T4 replacement therapy were enrolled in the study. All women with HT had positive TPOAbs. Forty healthy females negative for TPOAbs comparable for age and body mass index (BMI) participated in the study as controls. Exclusion criteria were a history of any acute or chronic disease, use of any medications (including oral contraceptives and vitamin supplements), smoking, alcoholism.

**Results:**

TPOAbs titers were higher in both groups of HT patients versus the healthy controls. Hcy levels were found to be significantly lower in treated HT patients (Me 11 μmol; IQR 4.2 μmol) as compared with healthy controls (Me 13.35 μmol; IQR 6.34 μmol; p = 0.0179). In contrast, no significant difference was found between non treated HT and control group in Hcy level. The study groups and the controls did not differ in age and BMI. Furthermore, levels of TSH, FT4, TC, LDL, HDL and TAG did not differ between the study group and the control group.

**Conclusion:**

The main finding of the study is a decrease in Hcy level in treated HT as compared with healthy controls. Based on our observations one can also assume that correct L-T4 replacement was associated here with a decrease of Hcy. Furthermore, it seems that non treated HT in euthyroidism is not associated with Hcy increase, in contrast to overt hypothyroidism. This may be just another argument against the concepts about the role of “euthyroid HT” in the development of atherosclerosis.

## Background

Homocysteine (Hcy) is a sulfur-containing amino acid naturally found in human blood and its metabolism is based on two divergent pathways: transsulfuration and remethylation [1]. Hcy has been investigated as a risk factor for cardiovascular disease since 1969, when McCully observed that two patients with homocystinuria were affected with extensive atherosclerosis and arterial thrombosis [2]. Since the association between elevated level of Hcy and increased risk of coronary heart disease was established [3,4], the issue whether there is a causal relation still remains unclear [5,6].

To date, hypothyroidism is considered as an independent risk factor for atherosclerosis. However, the atherogenic lipid profile does not fully explain the increased cardiovascular morbidity in hypothyroid individuals. For that reason, the possible association between hypothyroid state and Hcy concentration was suggested. Interestingly, increased Hcy level in overt hypothyroidsm was found in many studies [7-9]. Additionally, normalization of Hcy level was achieved after euthyroidism restoration [10]. In sharp contrast with the above mentioned results, decreased Hcy concentration was found in hyperthyroidism [11].

The possible mechanism responsible for increased Hcy level in hypothyroidism also remains a matter of recent debate. Firstly, the observed hyperhomocysteinemia may reflect impaired renal Hcy clearance. Hypothyroidism probably reduces glomerular filtration rate leading to increased creatinine and Hcy levels [12-14]. Secondly, impaired liver metabolism of Hcy linked with hypothyroidism may contribute to hyperhomocysteinemia. Decreased activity of both enzymes, methionine synthase and methylenetetrahydrofolate reductase was established in thyroidectomized rats and may also explain the elevated level of Hcy in hypothyroidism [15-17].

In contrast to overt thyroid disorders, data concerning Hcy concentration among patients with subclinical hypothyroidism (SH) is contradictory. Some studies showed that, despite atherogenic lipid profile in SH, Hcy level is not increased [18,19]. On the other hand, Hcy concentration was reported to be higher as compared with euthyroid controls [20].

In view of those controversies, and considering the fact that numerous thyroid disorders are caused by autoimmune disturbances, we hypothesized that it might be the autoimmunity against thyroid gland that initially affects Hcy production, even in pre-clinical phases of thyroid disorders. Therefore, the aim of this study was to evaluate Hcy level in context of anti-thyroperoxidase antibodies (TPOAbs) rather than thyroid function. To achieve this goal, Hcy concentration was determined in otherwise healthy and euthyroid women with, and without chronic autoimmune thyroiditis. The criterion of euthyroidism let us exclude the possible influence of thyroid dysfunction *per se* on Hcy concentrations.

## Methods

Thirty one euthyroid women treated with levothyroxine (L-T4) due to Hashimoto thyroiditis (HT) at the outpatient clinic of the Department of Endocrinology, Metabolism and Internal Medicine and twenty six females with chronic autoimmune thyroiditis in euthyroidism without L-T4 replacement therapy were enrolled in the study (Table 1). All women with HT had positive TPOAbs. Forty healthy females negative for TPOAbs comparable for age and body mass index (BMI) participated in the study as controls (Table 1).

**Table 1:** Characteristics of the study groups and the controls

	**Non treated Hashimoto (n = 26)**	**Treated Hashimoto (n = 31)**	**Controls (n = 40)**	**p**
Age (yr)	43 (17)	38 (18)	35.5 (15)	ns
BMI (kg/m^2^)	22.8 (3.6)	22.6 (4)	21.8 (5.40)	ns
TSH (mU/L)	1.64 (2.08)	2.07 (3.14)	1.54 (1.5)	ns
FT4 (pmol/L)	14.7 (2.27)^a^	17.13 (5.11)^b^	15.52 (2.23)^a,b^	p = 0.0019
TPOAbs (IU/mL)	242 (290)^b^	300 (391)^b^	9 (5.5)^a^	p < 0.0001
Hcy (μmol)	11 (4.2)^a,b^	10.8 (6.9)^a^	13.35 (6.34)^b^	p = 0.0179
[Mean ± SD]	[10.33 ± 3.36]^a,b^	[9.84 ± 4.24]^a^	[12.97 ± 6.71]^b^	
TC (mg/dl)	207 (64)	205 (60)	196 (51)	ns
LDL (mg/dl)	118.5 (50.6)	121 (45.6)	110.9 (37.4)	ns
HDL (mg/dl)	57 (30)	67 (16)	65 (15.5)	ns
TAG (mg/dl)	75 (38)	83 (47)	82 (46)	ns

Results are expressed as medians, followed by interquartile ranges given in brackets.

a,b – values followed by the same letters do not differ significantly at p < 0.05.

All subjects and controls were euthyroid, either spontaneously, or under L-T4 medication. None of the patients and the controls had a history of any acute or chronic disease, including diabetes mellitus, hypertension, angina pectoris, evidence of any kidney or liver disorder. Exclusion criteria were also use of any medications (including oral contraceptives and vitamin supplements), smoking, alcoholism.

All subjects underwent physical examination with recording of height, weight, heart rate, systolic and diastolic blood pressure. Blood samples were obtained after an overnight fast. In patients with HT blood samples were drawn before the ingestion of usual morning L-T4 medication. Serum levels of thyrotropin hormone (TSH), free thyroxine (FT4), TPOAbs, Hcy, total cholesterol (TC), low-density lipoprotein (LDL), high-density lipoprotein (HDL), triacylglycerol (TAG) were evaluated.

TSH and FT4 were measured using the electrochemiluminescence technique in Cobas E 601 (norm ranges: TSH 0.27–4.2 mU/l; FT4 11.5–21.0 pmol/l). TPOAbs were measured by radioimmunoassay (norm range: <34 IU/ml).

Serum Hcy levels were assessed by high-performance liquid chromatography (HPLC). The analyzed plasma thiol compounds (Hcy, Fluka Germany) were diluted with water at 2:1 ratio and reduced using 1% TCEP (Tris-(2-carboxyethyl)-phosphin-hydrochloride; Applichem, Germany) at 1:9 ratio. Subsequently, the sample was deproteinized using 1 M HClO_4_ (at 2:1 ratio) and applied to the HPLC/EC system.

To determine thiol concentration the samples were fed to the HPLC system (P580A; Dionex, Germany) coupled to an electrochemical detector (CoulArray 5600; ESA, USA). The analysis was performed in Termo Hypersil BDS C18 column (250 mm × 4.6 mm × 5 μm) (Germany) in isocratic conditions, using the mobile phase of 0.15 M phosphate buffer, pH 2.9, supplemented with 12.5-17% acetonitrile. The system was controlled, and the data were collected and processed using Chromeleon software (Dionex, Germany).

The study was approved by the Poznan University of Medical Sciences Ethical Commitee, and informed consent was signed by every subject.

Comparison of analyzed parameters between three groups (HT patients versus healthy controls) was performed by Kruskal-Wallis test with Dunn’s post-hoc tests because data did not follow normal distribution. Normality was analyzed by Shapiro-Wilk’s test. Results were presented as medians and interquartile ranges (IQR). Spearman’s correlation coefficient was used to measure the strength of relationship of analyzed parameters. All tests were performed two-tailed and were considered as significant at p < 0.05. Statistical analyses were performed with Statistica 10 (StatSoft Inc., Poland) and MedCalc version 10.3.2 (MedCalc Software, Mariakerke, Belgium) .

## Results

Descriptive characteristics for the study groups and the control group are shown in Table 1.

**Figure 1:**
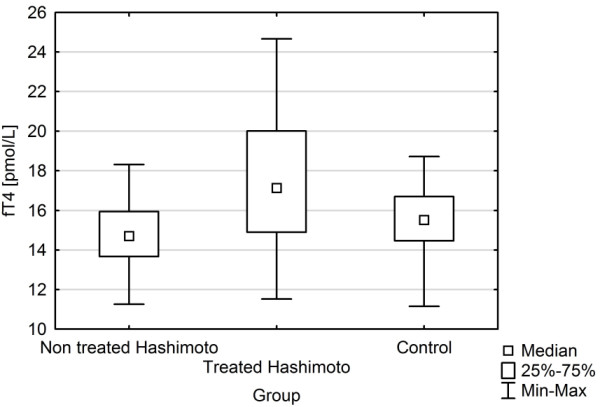
FT4 concentrations in non treated and treated HT and in controls.

**Figure 2:**
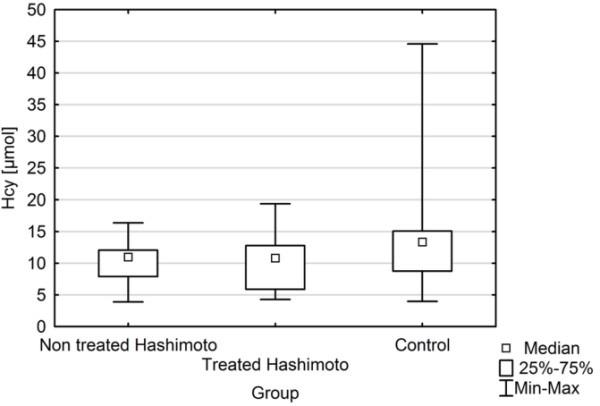
Hcy levels in non treated and treated HT and in controls.

The study groups and the controls did not differ in age and BMI.TSH levels of treated and non treated HT patients were similar to those of control subjects. FT4 concentrations of treated HT patients were significantly higher as compared with non treated HT and did not differ from controls (Figure 1). TPOAbs titers were higher in both groups of HT patients versus the healthy controls.Hcy levels were found to be significantly lower in treated HT patients as compared with healthy controls (Figure 2). In contrast, no significant difference was found between non treated HT and control group in Hcy level.

In treated HT females Hcy level was negatively correlated with age (p = 0.017289; r = -0.42). In contrast, correlation between Hcy and age was not found in non treated HT and in controls. No significant associations were found between Hcy and BMI, waist circumference, TSH, FT4, TPOAbs in all of HT patients and in healthy females. Furthermore, levels of TC, LDL, HDL and TAG did not differ between the study groups and the control group.

## Discussion

In this study, we investigated the influence of thyroid autoimmunity on Hcy concentrations. To achieve this goal, we used very strict criteria of enrollment, and we excluded the possible influence of hypothyroidism by including only patients who were euthyroid, obviously either spontaneously, or under medication. With this novel approach, we came to somewhat surprising conclusions that we discuss beneath.

We showed here that non treated HT and treated HT females had comparable TPOAbs, however treated HT group had higher level of FT4. Moreover, FT4 in treated HT and healthy controls did not differ indicating sufficient replacement therapy. The main finding of the study is a decrease in Hcy level in treated HT as compared with healthy controls.

Obviously, our findings should be understood in a broader context of the association between thyroid autoimmunity and atherosclerosis. Indeed, some authors suggest that chronic autoimmune thyroiditis per se may be considered as a novel risk factor of atherosclerosis independent of thyroid function [21-24]. Currently, however, the causative relationship between thyroid autoimmunity and increased risk of atherosclerosis has not yet been established. A few studies which were addressing this question investigated the effect of thyroid autoimmunity on lipid profile, abdominal obesity, fasting glucose and homeostasis model assessment (HOMA) insulin resistance, as well as carotid intima-media thickness (CIMT) [25-27]. However, in contrast to our study group, the study populations were not uniform, therefore the conclusions of these investigations are not comparable. Tamer et al. found that euthyroid HT (n = 84) patients had higher LDL level as compared with controls (n = 150) (p = 0.0042) [25]. Moreover, TPOAbs level was negatively correlated with HDL (p = 0.031; r = -0.137) and positively with TAG (p = 0.013; r = 0.158) and waist circumference (p = 0.048; r = 0.128). Ciccone et al. established that overweight or obese women with HT have increased IMT as compared with controls [27]. They suggested that the autoimmunity in HT patients is an independent factor that might accelerate atherosclerosis. However, they also found that HT patients had higher TSH levels and lower FT3.

It must be emphasized here that the effect of both hypothyroidism and hyperthyroidism on Hcy concentration has been investigated in many studies before. However, data concerning the impact of thyroid autoimmunity in euthyroid state on Hcy level is lacking.

To our knowledge, there was only one report of Hcy in context of thyroid autoimmunity in euthyroid premenopausal females with Hashimoto thyroiditis recently reported by Topaloglu et al. [26]. In this research, the study population was divided into two subgroups: first with TSH ≤ 2.5 IU/L and second with TSH > 2.5 IU/L. The controls were age-matched. CIMT was the only one evaluated parameter that was significantly higher in the study group regardless of TSH. CIMT was positively correlated with antithyroglobulin antibodies (p = 0.014; r = 0.328). In contrast to our results, Hcy level did not differ between the study and the control groups. However, in our study the control group had comparable BMI with the study group, therefore the body weight did not have any effect on the Hcy comparison. In contrast, Topaloglu et al. had a control group with BMI much lower than the study group (p < 0.001). Therefore, the potential influence of this co-variable on the Hcy analysis should be taken into consideration. New insight into the discussed problem was given in recently published research concerning Hcy level in patients with atrophic glossitis or burning mouth syndrome and autoimmune thyroiditis [28]. Wang et al. found increased Hcy level in these patients independently of thyroid function. Majority of the studied group positive for antithyroid antibodies (anti-thyroglobulin antibodies or anti-thyroid microsomal antibodies) was euthyroid. However, despite normal thyroid function in 85.8% of investigated individuals abnormal high blood Hcy level was established. Authors suggested that this finding was linked with vitamin B12 deficiency confirmed among this group.

Our study has some strengths and limitations. The main limitation of the present study is the number of patients who underwent the evaluation. However, it should be underlined, that all subjects were euthyroid females without any comorbidities and they did not take any medication. The only one difference between treated HT females and controls were TPOAbs. In our opinion, the homogeneity of subjects examined was the strength of this work that could balance its limitations.

Serum FT4 concentration is considered as an independent determinant of Hcy level [29]. As was mentioned above, Hcy level is generally decreased in hyperthyroid patients in contrast to hypothyroid subjects, who have higher Hcy concentration. Moreover, restoration of thyroid function leads to normalization of Hcy concentration. In general, our study and control groups were euthyroid, but this state was achieved by L-T4 replacement therapy in a group of treated HT women. This group had lower Hcy levels than normal controls, despite similar FT4 levels. A possible explanation of this finding is the fact that, in spite of similar hormone concentrations, these patients differed in that had different sources of thyroxine: it was endogenous in one group, and exogenous in the other. In our opinion, there is a causative relationship between L-T4 replacement therapy and decreased Hcy levels. Patients who are on L-T4 therapy have daily changes of FT4 serum concentration that result from pharmacokinetic properties of this medication. The maximum FT4 concentration occurs approximately two hours after the drug ingestion [30]. Moreover, there is a transient increase of FT4 serum level after ingestion of L-T4 for 5 hours [31]. Since FT4 directly influences Hcy concentration, during this time Hcy metabolism is similar to hyperthyroid state and it may lead to decreased Hcy levels in treated HT patients in contrast to healthy controls, in whom FT4 output is adjusted to real needs and the rate of physiological elimination .

## Conclusions

The main finding of the study is a decrease in Hcy level in treated HT as compared with healthy controls. Our study adds further evidence to the debate on possible association between chronic autoimmune thyroiditis and atherosclerosis. It seems that non treated HT in euthyroidism is not associated with Hcy increase, in contrast to overt hypothyroidism. This may be just another argument against the concepts about the role of “euthyroid HT” in the development of atherosclerosis, which is of quite importance considering the high prevalence of high TPOAbs titers in Europe. Furthermore, that Hcy was lower in the treated group may point to the beneficial role of L-T4 treatment in general. Correct L-T4 replacement was associated here with a decrease of Hcy. In conclusion, L-T4 treatment may further add to medical approach aimed at atherosclerosis risk reduction with regard to Hcy decrease in L-T4-treated women with chronic autoimmune thyroiditis. This last concept, however, as drawn only from a cross-sectional setting, requires further investigation in observational studies.

## Competing interests

The authors declare that there are no conflicts of interests.

## Authors’ contributions

MO conceived of the study, and participated in its design and coordination and recruitment of patients, drafted the manuscript; JD participated in design of the study, carried out the high-performance liquid chromatography and helped to draft the manuscript; NSG collected and analyzed the data, prepared the manuscript, performed the statistical analysis and the literature review; AO carried out the immunoassays and high-performance liquid chromatography; MKO participated in design of the study and coordination; MM performed the statistical analysis, helped to draft the manuscript; JF participated in design of the study and recruitment of patients; WK corrected the manuscript, approved the final version of the manuscript; MR corrected the manuscript, approved the final version of the manuscript. All authors read and approved the final manuscript.

## Pre-publication history

The pre-publication history for this paper can be accessed here:

http://www.biomedcentral.com/1472-6823/14/18/prepub

## References

[B1] Selhub J Homocysteine metabolism Annu Rev Nutr 1999 19 217 246 10.1146/annurev.nutr.19.1.217 10448523

[B2] McCully KS Vascular pathology of homocysteinemia: implications for the pathogenesis of arteriosclerosis Am J Pathol 1969 56 111 128 5792556 PMC2013581

[B3] Homocysteine Studies Collaboration Homocysteine and risk of ischemic heart disease and stroke: a meta-analysis JAMA 2002 288 2015 2022 10.1001/jama.288.16.2015 12387654

[B4] Klerk M Verhoef P Clarke R Blom HJ Kok FJ Schouten EG MTHFR Studies Collaboration Group MTHFR 677C → T polymorphism and risk of coronary heart disease: a meta-analysis JAMA 2002 288 2023 2032 10.1001/jama.288.16.2023 12387655

[B5] Thampi P Stewart BW Joseph L Melnyk SB Hennings LJ Nagarajan S Dietary homocysteine promotes atherosclerosis in apoE-deficient mice by inducing scavenger receptors expression Atherosclerosis 2008 197 620 629 10.1016/j.atherosclerosis.2007.09.014 17950295

[B6] Clarke R Halsey J Bennett D Lewington S Homocysteine and vascular disease: review of published results of the homocysteine-lowering trials J Inherit Metab Dis 2011 34 83 91 10.1007/s10545-010-9235-y 21069462

[B7] Nedrebø BG Ericsson UB Nygård O Refsum H Ueland PM Aakvaag A Aanderud S Lien EA Plasma total homocysteine levels in hyperthyroid and hypothyroid patients Metabolism 1998 47 89 93 10.1016/S0026-0495(98)90198-6 9440483

[B8] Morris MS Bostom AG Jacques PF Selhub J Rosenberg IH Hyperhomocysteinemia and hypercholesterolemia associated with hypothyroidism in the third US National Health and Nutrition Examination Survey Atherosclerosis 2001 155 195 200 10.1016/S0021-9150(00)00537-2 11223442

[B9] Gunduz M Gunduz E Kircelli F Okur N Ozkaya M Role of surrogate markers of atherosclerosis in clinical and subclinical thyroidism Int J Endocrinol 2012 doi:10.1155/2012/109797 10.1155/2012/109797 PMC3296143 22505888

[B10] Lien EA Nedrebø BG Varhaugh JE Nygard O Aakvaag A Ueland PM Plasma total homocysteine levels during short-term iatrogenic hypothyroidism J Clin Endocrinol Metab 2000 85 1049 1053 10720038 10.1210/jcem.85.3.6439

[B11] Nedrebø BG Nygård O Ueland PM Lien EA Plasma total homocysteine in hyper- and hypothyroid patients before and during 12 months of treatment Clin Chem 2001 47 1738 1741 11514424

[B12] Diekman MJ van der Put NM Blom HJ Tijssen JG Wiersinga WM Determinants of changes in plasma homocysteine in hyperthyroidism and hypothyroidism Clin Endocrinol (Oxf) 2001 54 197 204 10.1046/j.1365-2265.2001.01170.x 11207634

[B13] Lien EA Nedrebø BG Varhaug JE Nygård O Aakvaag A Ueland PM Plasma tHcy levels during short-term iatrogenic hypothyroidism J Clin Endocrinol Metab 2000 85 1049 1053 10720038 10.1210/jcem.85.3.6439

[B14] Nedrebø BG Nygård O Ueland PM Lien EA Plasma tHcy in hyper- and hypothyroid patients before and during 12 months of treatment Clin Chem 2001 47 1738 1741 11514424

[B15] Chan MM Stokstad EL Metabolic responses of folic acid and related compounds to thyroxine in rats Biochimica Biophysica Acta 1980 632 244 253 10.1016/0304-4165(80)90082-3 7417525

[B16] Nair CP Viswanathan G Noronha JM Folate-mediated incorporation of ring-2-carbon of histidine into nucleic acids: influence of thyroid hormone Metabolism 1994 43 1575 1578 10.1016/0026-0495(94)90019-1 7990714

[B17] Ayav A Alberto JM Barbe F Brunaud L Gerard P Merten M Gueant JL Defective remethylation of homocysteine is related to decreased synthesis of coenzymes B2 in thyroidectomized rats Amino Acids 2005 28 37 43 10.1007/s00726-004-0151-z 15645165

[B18] Luboshitzky R Aviv A Herer P Lavie L Risk factors for cardiovascular disease in women with subclinical hypothyroidism Thyroid 2002 12 421 425 10.1089/105072502760043512 12097204

[B19] Atabek ME Pirgon O Erkul I Plasma homocysteine concentration in adolescents with subclinical hypothyroidism J Pediatr Endocrinol Metab 2003 16 1245 1248 14714746 10.1515/jpem.2003.16.9.1245

[B20] Sengul E Cetinarslan B Tarkun I Canturk Z Turemen E Homocysteine concentrations in subclinical hypothyroidism Endocr Res 2004 30 351 359 10.1081/ERC-200033558 15554351

[B21] Bastenie PA Vanhaelst L Golstein J Smets P Asymptomatic autoimmune thyroiditis and coronary heart disease.Cross-sectional and prospective studies Lancet 1977 2 155 158 69779 10.1016/s0140-6736(77)90177-5

[B22] Hak AE Pols HA Visser TJ Drexhage HA Hofman A Witteman JC Subclinical hypothyroidism is an independent risk factor for atherosclerosis and myocardial infarction in elderly women: the Rotterdam study Ann Intern Med 2000 132 270 278 10681281 10.7326/0003-4819-132-4-200002150-00004

[B23] Nyirenda MJ Clark DN Finlayson AR Read J Elders A Bain M Fox KA Toft AD Thyroid disease and increased cardiovascular risk Thyroid 2005 15 718 724 10.1089/thy.2005.15.718 16053389

[B24] Zoller B Li X Sundquist J Sundquist K Risk of subsequent ischemic and hemorrhagic stroke in patients hospitalized for immune-mediated diseases: a nationwide follow-up study from Sweden BMC Neurol 2012 12 41 10.1186/1471-2377-12-41 22708578 PMC3430565

[B25] Tamer G Mert M Tamer I Mesci B Kilic D Arik S Effects of thyroid autoimmunity on abdominal obesity and hyperlipideamia Endokrynol Pol 2011 62 421 428 22069103

[B26] Topaloglu O Gokay F Kucukler K Burnik FS Mete T Yavuz HC Berker D Guler S Is autoimmune thyroiditis a risk factor for early atherosclerosis in premenopausal women even if in euthyroid status? Endocrine 2013 44 145 151 10.1007/s12020-012-9842-5 23184180

[B27] Ciccone MM De Pergola G Porcelli MT Scicchitano P Caldarola P Lacoviello M Pietro G Giorgino F Favale S Increased carotid IMT in overweight and obese women affected by Hashimoto’s thyroiditis: an adiposity and autoimmune linkage? BMC Cardiovasc Disord 2010 10 22 10.1186/1471-2261-10-22 20509904 PMC2885992

[B28] Wang YP Lin HP Chen HM Kuo YS Lang MJ Sun A Hemoglobin, iron, and vitamin B12 deficiencies and high blood homocysteine levels in patients with anti-thyroid autoantibodies J Formos Med Assoc 2012 doi:10.1016/j.jfma.2012.04.003 10.1016/j.jfma.2012.04.003 24630032

[B29] Orzechowska-Pawilojc A Sworczak K Lewczuk A Babinska A Homocysteine, folate and cobalamin levels in hypothyroid women before and after treatment Endocr J 2007 54 471 476 10.1507/endocrj.K06-112 17464093

[B30] Colucci P Seng Yue C Ducharme M Benvenga S A review of the pharmacokinetics of levothyroxine for the treatment of hypothyroidism Eur Endocrinol 2013 9 40 47 10.17925/EE.2013.09.01.40 PMC6193522 30349610

[B31] Ain KB Pucino F Shiver TM Banks SM Thyroid hormone levels affected by time of blood sampling in thyroxine-treated patients Thyroid 1993 3 81 85 10.1089/thy.1993.3.81 8369656

